# Risk of Delayed Discharge and Reoperation of Gastric Bypass Patients with Psychiatric Comorbidity—a Nationwide Cohort Study

**DOI:** 10.1007/s11695-020-04483-7

**Published:** 2020-03-09

**Authors:** Ylva Trolle Lagerros, Lena Brandt, Magnus Sundbom, Jakob Hedberg, Robert Bodén

**Affiliations:** 1 Department of Medicine, Division of Clinical Epidemiology, Karolinska Institutet, T2, SE 171 76 Stockholm, Sweden; 2 Center for Obesity, Academic Specialist Center, Stockholm Health Services, Stockholm, Sweden; 3 Department of Surgical Sciences, Uppsala University, Uppsala, Sweden; 4 Department of Neuroscience Psychiatry, Uppsala University, Uppsala, Sweden

**Keywords:** Bariatric surgery, Length of stay, Mental health, Obesity, Patient readmission, Postoperative complications

## Abstract

**Background:**

Gastric bypass (GBP) surgery is considered a safe and effective treatment for obesity. However, there is uncertainty regarding the impact of preexisting psychiatric comorbidity on GBP complications. We have investigated whether a psychiatric diagnosis before GBP surgery is associated with delayed discharge (the odds of being in the 90th percentile of length of stay) and rate of reoperation in a nationwide Swedish cohort.

**Methods:**

Patients undergoing GBP surgery during 2008–2012 were identified and followed up through the National Patient Register and the Prescribed Drug Register. Logistic regression models were fitted to the studied outcomes.

**Results:**

Among the 22,539 patients identified, a prior diagnosis of bipolar disorder, schizophrenia, depression, neurotic disorders, ADHD (attention deficit hyperactivity disorder), substance use disorder, eating disorder, personality disorder, or self-harm since 1997 (*n* = 9480) was found to be associated with delayed discharge after GBP surgery (odds ratio [OR] = 1.47, confidence interval [CI] 1.34–1.62), especially in patients with psychiatric hospitalization exceeding 1 week in the 2 years preceding GBP surgery (OR = 2.06, CI 1.30–3.28), compared with those not hospitalized within psychiatry. Likewise, patients with a prior psychiatric diagnosis were more likely to be reoperated within 30 days (OR = 1.25, CI 1.11–1.41), with twice the likelihood OR 2.23 (CI 1.26–3.92) for patients with psychiatric hospitalization of up to a week in the 2 years preceding GBP surgery, compared with patients who had not been hospitalized within psychiatry.

**Conclusions:**

A psychiatric diagnosis before GBP surgery was associated with delayed discharge and increased likelihood of reoperation within 30 days. Patients with a prior psychiatric diagnosis may, therefore, need additional attention and support.

**Electronic supplementary material:**

The online version of this article (10.1007/s11695-020-04483-7) contains supplementary material, which is available to authorized users.

## Introduction

Obesity is increasing worldwide, including patients suffering from psychiatric disorders. The most effective treatment for obesity is bariatric surgery, which has been proven to be safe [1] and cost-effective [2].

Psychiatric disorders are prevalent among bariatric surgery candidates. In a study of more than 10,000 Canadian patients seeking bariatric surgery, 51% had a past or present psychiatric diagnosis, most commonly depression, which was found in 41.7% of the group [3]. Likewise, in the Longitudinal Assessment of Bariatric Surgery study, 38.7% of the patients interviewed had a lifetime history of major depressive disorder. Furthermore, 33.2% had a prior diagnosis of alcohol abuse or dependence [4]. In another North American sample, 6% of patients screened positive for bipolar symptoms preoperatively [5]. Symptoms of attention deficit hyperactivity disorder (ADHD) are more than twice as common in Swedish patients seeking bariatric surgery than in the general population, and are also significantly correlated with symptoms of anxiety, depression, and eating disorders [6]. In a recent meta-analysis, the prevalence of binge eating disorder was estimated to be 17%, and depression to be 19% in those undergoing bariatric surgery [7].

According to the general surgical literature, not only depression lowers the threshold for postoperative pain, but also it is a predictor of patient dissatisfaction and increased postoperative mortality [8]. Moreover, psychiatric comorbidity may be a risk factor for somatic complications after surgery [9]. At the same time, there seem to be bi-directional relationships between surgical complications and higher depression and lower physical quality-of-life scores [10]. Nonetheless, a history of psychiatric comorbidity is not a contraindication for bariatric surgery, while severe active psychiatric symptoms may be [11].

Hospital readmission has become a quality indicator in bariatric surgery, and reducing readmission will both lower costs and improve health care. There is an increased risk of hospitalization during the first 2 years after gastric bypass (GBP) surgery in patients with a previous diagnosis of self-harm or depression [12]. Furthermore, patients with psychiatric comorbidity show a higher frequency of emergency department visits up to 2 years after bariatric surgery [13]. This patient group is also at a greater risk of 30-day readmission after bariatric surgery [14]. However, few studies have been carried out on the delayed discharge of patients with psychiatric comorbidity undergoing bariatric surgery. There is also a lack of data on the risk of reoperation in this patient group.

The purpose of the present study was thus to investigate possible associations between psychiatric diagnoses and delayed discharge (i.e., the odds of being in the 90th percentile of length of stay), and the odds of reoperation within 30 days of GBP surgery in a nationwide Swedish cohort.

## Methods

To conduct this nationwide retrospective cohort study, we were given access to the Swedish National Patient Register by the Swedish National Board of Health and Welfare. This register contains diagnoses from hospital in- and outpatient care and covers more than 99% of all somatic and psychiatric discharges [15]. Psychiatric diagnoses are made by medical doctors and recorded following all contacts with specialized health care in Sweden. Reporting to the register is mandatory in Sweden, also for private clinics, and thus, there is a high degree of completeness.

Most patients with psychiatric illnesses are treated within specialized psychiatric in- or outpatient services in Sweden; thus, these patients will be identified through the National Patient Register. However, only 19% of those who present with major depression and 18% of those who present with drug abuse in Swedish primary care also present in specialist or inpatient care [16]. Since the National Patient Register does not cover diagnoses made by doctors in primary care and since almost 80% of those diagnosed with major depression in primary care are prescribed antidepressants [16], we included patients with at least one filled prescription of an antidepressant (according to the Anatomical Therapeutic Chemical classification system, ATC N06A) from 2005 and onwards. This is a more sensitive marker of depression, although less specific, since antidepressants can also be used for other neurotic indications. Similarly, substance use disorder (SUD) and its treatment were identified through the ICD diagnosis codes F10–F19 (SUD of alcohol, opioids, cannabis, hypnotics, cocaine, hallucinogens, and psychoactive drugs), as well as through the ATC codes for N07BB drugs (disulfiram, acamprosate, naltrexone) and N07BC drugs (buprenorphine and methadone).

Data on dispensed antidepressants and treatment for SUD were collected from the Swedish Prescribed Drug Register, which is a nationwide individual-based register of dispensed prescription drugs, established in 2005, and coded according to the ATC classification system [17]. It was possible to link data for each patient in the two databases through the unique personal identity number used in Sweden [18].

In 2012, 12% of the Swedish population was obese according to Statistics Sweden, but only 78 per 100,000 inhabitants were deemed eligible for bariatric surgery. This type of surgery was conducted at 41 centers around Sweden, the vast majority of procedures being carried out in public hospitals [19]. Patients undergoing GBP surgery from 2008 to 2012 were identified by the procedure codes for gastric bypass surgery (JDF10) and for laparoscopic gastric bypass surgery (JDF11) (as defined in the Swedish version of the Nordic Medico-Statistical Committee classification of surgical procedures, version 1.9). A diagnosis of obesity was identified through the code E66 (International Statistical Classification of Diseases ICD-10). Gastric banding and gastric sleeve surgery were excluded, as they constituted only 0.77% and 0.72%, respectively, of all bariatric procedures in Sweden during this period [19]. We also used procedure codes to identify patients who underwent reoperation for complications such as bleeding, leakage, and gastrointestinal obstruction, within the first 30 days of their first GBP surgery.

All patients underwent presurgical psychological evaluation and were assessed with regard to psychiatric multimorbidity before bariatric surgery, although the thoroughness of this evaluation may have differed between bariatric surgery centers. To quantify psychiatric multimorbidity, we identified any psychiatric diagnosis recorded in the National Patient Register before GBP surgery, based on ICD-10 diagnoses at inpatient or specialized psychiatric outpatient services. The following ICD diagnoses were used: bipolar disorder (F30–F31), schizophrenia spectrum disorders (F20), depression (F32–F33), neurotic disorders (F40 agoraphobia, F41 anxiety disorders, F42 obsessive-compulsive disorder, F43 reaction to severe stress, including posttraumatic stress syndrome and adjustment disorders, F44 dissociative and conversion disorders, F45 somatoform disorders, F48 other non-psychotic mental disorders), attention deficit hyperactivity disorder (ADHD) (F90.0), eating disorders (F50), personality disorder (F60–F61), and self-harm (X60–X84, Y10–Y34).

Due to the small number of patients diagnosed with schizophrenia, these were combined with those suffering from bipolar disorder to form a broader diagnostic group called severe mental illness. Since depression and bipolar disorder are never diagnosed in the same person at the same time, while depression is a necessary component of bipolar disorder, individuals can be misdiagnosed as having depression, rather than as having bipolar disorder. Likewise, those with schizophrenia can initially be incorrectly diagnosed as having depression. Therefore, if a patient with severe mental illness also had a diagnosis of depression, the patient was coded as having severe mental illness.

### Statistical Analyses

Frequencies and percentages were calculated for categorical data, and means and standard deviations (SD) were used to describe continuous variables for the baseline characteristics of the cohort. Logistic regression models were fitted, and the odds ratio (OR) and 95% confidence intervals (CIs) for delayed discharge and reoperation within 30 days of GBP surgery were determined.

Crude and adjusted ORs were calculated for each diagnostic group. The comparison group consisted of GBP surgery patients with no recorded psychiatric diagnosis and not taking any antidepressant medication since 2005. We also determined whether hospitalization for 0–7 days, or 8 or more days within psychiatry in the 2 years preceding GBP surgery was associated with delayed discharge or reoperation within 30 days, compared to patients without psychiatric hospitalization in the 2 years preceding GBP surgery. As a sensitivity analysis, we only included the 2 years preceding GBP surgery and calculated crude and adjusted ORs for each diagnostic group, and compared these with those of patients with no recorded psychiatric diagnosis taking no antidepressant medication.

Due to the apparent risk of changes in surgical procedures over time, all analyses were conditioned on calendar year. In the most adjusted analyses, we furthermore controlled for gender; age categorized into three roughly equally sized groups, i.e., < 35, 35–44, 45+; type of GBP surgery (open or laparoscopic); and other psychiatric diagnoses as categorical variables. This adjustment strategy allowed comorbid diagnoses to have different influences in different diagnostic groups.

All analyses were performed using SAS version 9.4 (SAS Institute Inc., Cary, NC).

## Results

During the 5-year study period, 22,539 subjects underwent GBP surgery in Sweden. The mean duration of follow-up after GBP surgery was 546 days. The majority (75.3%) were women (Table 1). Of the entire sample, 42% had a psychiatric diagnosis and/or were being treated with antidepressants, 1286 (5.7%) had two or more of the psychiatric diagnoses considered, and 1.5% had been hospitalized due to psychiatric disorders during the 2 years preceding GBP surgery. The average length of stay postsurgery decreased during the study period, from an average of 4.3 days in 2008 to 2.3 days in 2012 (*p* < 0.05). The 90th percentile of length of stay decreased from > 6 to > 4 days during the same time period (*p* = 0.01).

**Table 1:** Characteristics of subjects who underwent gastric bypass surgery in Sweden between 2008 and 2012

	Number	Percentage
Number of subjects	22,539	
Gender		
Male (%)	5578	24.7
Female (%)	16,961	75.3
Age at surgery (years), mean (SD) 41.3 (11.0)		
< 35	6204	27.5
35–44	7465	33.1
> 44	8870	39.4
Type of surgery		
Open gastric bypass surgery (%)	1565	6.9
Laparoscopic gastric bypass surgery (%)	20,974	93.1
Days of follow-up postsurgery		
Mean (SD) 546.0 (233.1)		
Min–max 2–730		
Year of surgery		
2008 (%)	2270	10.1
2009 (%)	3609	16.0
2010 (%)	4742	21.0
2011 (%)	6420	28.5
2012 (%)	5498	24.4
Psychiatric hospitalization during the 2 years prior to surgery		
None (%)	22,205	98.5
0–7 days (%)	139	0.6
> 8 days (%)	195	0.9
Psychiatric diagnosis during the 2 years prior to surgery	2602	11.5

In the most adjusted model, the discharge of patients with a psychiatric diagnosis (*n* = 4856) was more likely to be delayed after GBP surgery than that of patients without a psychiatric diagnosis (OR 1.62; CI 1.44–1.82). A previous diagnosis of eating disorder almost doubled the likelihood of delayed discharge after surgery, compared to patients without a psychiatric diagnosis or taking antidepressant medication (OR 1.97; CI 1.04–3.71). A diagnosis of depression (or antidepressant medication), neurotic disorder, and SUD or treatment for SUD, diagnosed since 1997, also significantly increased the likelihood of delayed discharge, compared to those without such diagnoses or treatments (Table 2). A diagnosis of SUD or treatment for SUD, or ADHD in the 2 years preceding GBP surgery increased the likelihood of delayed discharge (adjusted OR 2.49, CI 1.55–4.00; and OR 2.15, CI 1.16–3.99, respectively), and a diagnosis of personality disorder in the 2 years preceding GBP surgery more than tripled the adjusted OR to 3.23 (CI 1.69–6.17), compared to patients without a psychiatric diagnosis or antidepressant medication (Supplementary Table 1). Furthermore, psychiatric hospitalization exceeding a week in the 2 years preceding GBP was associated with a doubled likelihood of delayed discharge after surgery (adjusted OR 2.06; CI 1.30–3.28), compared to patients who had not been hospitalized within psychiatry in the 2 years preceding GBP surgery (data not shown).

**Table 2.** Associations between psychiatric diagnoses and delayed discharge after gastric bypass surgery, odds ratios (OR) with 95% confidence intervals (CI)

	Total	Hospital stay ≥ 90th percentile of number of postoperative days in somatic ward^d^		Crude OR conditioned on calendar year (95% CI)	Model 1^b^ Adjusted OR (95% CI)	Model 2^c^ Adjusted OR (95% CI)
	Number	Number	%			
All	22,539	2215	9.8			
None of the below mentioned diagnoses and no antidepressant medication prescribed since 2005	13,059	1118	8.6	REF 1.00	REF 1.00	REF 1.00
Diagnosis below or antidepressant medication	9480	1097	11.6	1.43 (1.31–1.56)	1.42 (1.30–1.56)	1.47 (1.34–1.62)
Diagnosis below						
Diagnoses since 1997						
Severe mental illness (Bipolar disorder/ Schizophrenia)^a^	375	42	11.2	1.55 (1.10–2.17)	0.96 (0.52–1.78)	0.92 (0.48–1.75)
Depression ^a, e^	2100	275	13.1	1.68 (1.45–1.94)	1.38 (1.11–1.71)	1.43 (1.13–1.80)
Neurotic disorders ^f^	2769	358	12.9	1.68 (1.47–1.91)	1.48 (1.23–1.78)	1.47 (1.21–1.79)
Attention deficit hyperactivity disorder	314	44	14.0	1.99 (1.42–2.79)	1.51 (0.82–2.79)	1.58 (0.83–3.00)
Substance use disorder or treatment for substance use disorder	971	135	13.9	1.79 (1.47–2.18)	1.39 (1.02–1.91)	1.41 (1.01–1.97)
Eating disorder	285	39	13.7	1.76 (1.23–2.50)	1.72 (0.93–3.16)	1.97 (1.04–3.71)
Personality disorder	551	77	14.0	1.82 (1.41–2.36)	1.29 (0.68–2.45)	1.20 (0.60–2.37)
Self-harm	1169	154	13.2	1.57 (1.31–1.89)	1.16 (0.87–1.55)	1.25 (0.92–1.69)
None of the above, but antidepressant medication prescribed after year 2005^g^	4624	497	10.7	1.31 (1.17–1.46)	1.24 (1.10–1.39)	1.31 (1.16–1.48)

^a^In case of diagnoses of both severe mental illness and depression, severe mental illness was chosen.

^b^Adjusted for age, sex, conditioned on calendar year.

^c^Adjusted for age, sex, type of gastric bypass surgery, the other diagnoses since 1997 (including treatment for SUD, but not antidepressant medication) (reviewer #1, comment #4) and conditioned on calendar year.

^d^≥ 90th percentile of number of postoperative days was ≥6 days in 2008–2009, ≥5 days in 2010, and ≥4 days in 2011–2012.

^e^Does not include the group prescribed antidepressants with no diagnosis of depression.

^f^Agoraphobia, anxiety disorders, obsessive-compulsive disorder, reaction to severe stress including post-traumatic stress syndrome, adjustment disorders, dissociative and conversion disorders, somatoform disorders, other nonpsychotic mental disorders (reviewer #2, comment #5)

^g^Filled prescription from the pharmacotherapeutic group N06A (according to the Anatomical Therapeutic Chemical classification system, ATC).

An increased likelihood of reoperation within the first 30 days was found among patients with a psychiatric diagnosis prior to GBP surgery, compared with that among patients without a psychiatric diagnosis (adjusted OR 1.37; CI 1.19–1.57) (Table 3). However, patients recently diagnosed with SUD or receiving treatment for SUD, or diagnosed with personality disorder in the 2 years preceding surgery had more than doubled odds of reoperation within the first 30 days (adjusted OR 2.22, CI 1.27–3.87; and 2.28, CI 1.03–5.05, respectively), compared with patients without a psychiatric diagnosis. A recent diagnosis of eating disorder (within 2 years before surgery) increased the odds of reoperation within the first 30 days more than threefold, compared to patients without a psychiatric diagnosis (adjusted OR 3.17; CI 1.21–8.29) (Supplementary Table 2). Psychiatric hospitalization of up to a week in the 2 years preceding GBP surgery was also associated with higher reoperation within 30 days (adjusted OR 2.23; CI 1.26–3.92), compared to patients who had not been hospitalized within psychiatry in the 2 years preceding GBP surgery (data not shown).

**Table 3:** Associations between psychiatric diagnoses and reoperation within 30 days of gastric bypass surgery, odds ratios (ORs) with 95% confidence intervals (CIs)

	Total	Reoperation within 30 days		Crude OR conditioned on calendar year (95% CI)	Model 1^b^ Adjusted OR (95% CI)	Model 2^c^ Adjusted OR (95% CI)
	Number	Number	%			
All	22,539	1270	5,6			
None of the below mentioned diagnoses and no antidepressant medication prescribed since 2005	13,059	667	5.1	Ref 1.00	Ref 1.00	Ref 1.00
Diagnosis below or antidepressant medication	9480	603	6.4	1.26 (1.13–1.41)	1.26 (1.12–1.41)	1.25 (1.11–1.41)
Diagnosis below	4856	327	6.7	1.34 (1.17–1.54)	1.39 (1.21–1.59)	1.37 (1.19–1.57)
Diagnoses since 1997						
Severe mental illness (Bipolar disorder/Schizophrenia)^a^	375	25	6.7	1.34 (0.89–2.03)	1.47 (0.77–2.80)	1.45 (0.76–2.78)
Depression^a,d^	2100	145	6.9	1.38 (1.15–1.67)	1.28 (0.97–1.69)	1.28 (0.97–1.69)
Neurotic disorders^e^	2769	187	6.8	1.35 (1.14–1.60)	1.37 (1.09–1.73)	1.35 (1.06–1.70)
Attention deficit hyperactivity disorder	314	15	4.8	0.97 (0.57–1.64)	0.91 (0.35–2.36)	0.88 (0.33–2.32)
Substance use disorder or treatment for substance use disorder	971	70	7.2	1.46 (1.13–1.88)	1.46 (0.98–2.16)	1.46 (0.98–2.17)
Eating disorder	285	15	5.3	1.03 (0.61–1.74)	1.04 (0.43–2.55)	1.08 (0.44–2.62)
Personality disorder	551	43	7.8	1.59 (1.15–2.19)	1.48 (0.69–3.17)	1.41 (0.66–3.03)
Self-harm	1169	79	6.8	1.34 (1.05–1.70)	1.25 (0.87–1.80)	1.29 (0.89–1.86)
None of the above, but antidepressant medication prescribed after year 2005^f^	4624	276	6.0	1.18 (1.02–1.36)	1.11 (0.96–1.29)	1.11 (0.96–1.29)

^a^In case of diagnoses of both severe mental illness and depression, severe mental illness was chosen

^b^Adjusted for age and sex, and conditioned on calendar year

^c^Adjusted for age, sex, type of gastric bypass surgery, and the other diagnoses since 1997 (including treatment for SUD, but not antidepressant medication) and conditioned on calendar year

^d^Does not include the group prescribed antidepressants with no diagnosis of depression

^e^Agoraphobia, anxiety disorders, obsessive-compulsive disorder, reaction to severe stress including posttraumatic stress syndrome, adjustment disorders, dissociative, and conversion disorders, somatoform disorders, and other non-psychotic mental disorders

^f^Filled prescription from the pharmacotherapeutic group N06A (according to the Anatomical Therapeutic Chemical (ATC) classification system)

## Discussion

In this nationwide GBP surgery cohort, delayed discharge was more likely in the patient group with a psychiatric diagnosis, in particular patients with prior diagnoses of eating disorders, SUD, neurotic disorder, or depression, than that in patients without a psychiatric diagnosis. Moreover, we found an increased likelihood of reoperation during the first month after GBP among patients with a recent diagnosis (in the 2 years preceding GBP) of eating disorders, SUD, or personality disorder, compared with that among patients without psychiatric diagnoses or not taking antidepressant medication before GBP surgery.

A US study has shown that patients with anxiety disorder have a higher 30-day readmission rate than controls [20]. Others have reported that patients with major depressive disorder/bipolar disorder are at a higher risk of readmission than patients with no such disorder [14]. Patients with depression and anxiety have also been reported to have more emergency department visits during the first 3 months after bariatric surgery [13]. However, emergency department visits or readmission do not necessarily lead to reoperation.

The literature regarding delayed discharge and reoperations in GBP patients with psychiatric comorbidity is sparse. Jalilvand et al. reported that patients with depression and anxiety had a higher incidence of long hospitalization after bariatric surgery than those without preexisting psychiatric diagnoses [20]. Our observation that the discharge of patients with an eating disorder or SUD was delayed is interesting, given recent theories that binge eating disorder may be an addictive disorder [21]. A compromised reward system could be a common factor in these disorders. The drastic change in eating behavior, and subsequent loss of intense rewards from eating, might lead to decreased motivation and slower mobilization, leading to the clinical decision to delay discharge.

Our finding that patients with neurotic disorders have a higher likelihood of delayed discharge is in line with previous observations that patients with high levels of anxiety have higher pain ratings and require more analgesia after GBP surgery [22]. This could be a potential reason for delayed discharge. Delayed hospital discharge has also been reported in patients with alcohol [23] and illicit drug abuse [24], which is in line with our results of delayed discharge after GBP surgery in the group with SUD. Our finding that a diagnosis of an eating disorder increased the likelihood of reoperation threefold already during the first month, has, to the best of our knowledge, not been reported before. However, a previous study of possible reasons for later revised bariatric surgery revealed that patients who underwent reoperation reported more non-normative eating patterns, such as binge eating and unhealthy food selection [25], stressing the importance of a healthy eating pattern after bariatric surgery.

Preoperative use of opioids before major abdominal surgery has been shown to increase length of stay, complications, and readmissions, compared to patients who did not use opioids before surgery [26]. Potential reasons could be complicated pain control, impaired gastrointestinal control increasing the likelihood of ileus, and increased risk of infection due to opioid-induced infectious complications, as demonstrated in US patients with traumatic injuries [27]. The group with SUD may also have difficulties following treatment recommendations, for example, practices for the prevention of surgical site infections. Patients with depression have been found to be less compliant with postoperative medical recommendations [13]. Likewise, the ability of manic patients to adhere to postsurgical guidelines could be undermined due to impulsivity and poor decision-making, which may also increase the risk of them not attending postsurgery appointments [28]. All these potential problems associated with suboptimal adherence to postoperative recommendations, including new eating habits, the daily need of supplements, and annual checkups, as well as refraining from seeking medical assistance if not feeling well, pose an overall increased risk to this group of patients. Thus, specialized follow-up programs may be needed, including close contact with psychiatric care.

The strengths and limitations of this study are those inherent in its registry-based design. Through access to nationwide, prospectively collected register information, with minimal loss to follow-up, we were able to study all patients undergoing GBP surgery during a 5-year period in Sweden. Misclassification of the exposures and outcomes was minimized through individual linkages between essentially complete, high-quality national register data, using the unique personal identity number assigned to each resident in Sweden. However, this process did not identify individuals who had a mental health condition, but had not yet been diagnosed or treated, or patients who had been diagnosed with depression by a primary care physician and only received, for example, cognitive behavioral therapy. It should also be noted that there is no information available in the National Patient Register on how the psychiatric diagnoses were established. However, some of the psychiatric diagnoses in the national Swedish registers, such as schizophrenia, bipolar disorder, and personality disorder, have been validated and found to have rather high positive predictive values [29, 30].

Although we conditioned on calendar year and controlled for potential confounders, such as other psychiatric diagnoses and treatment for SUD, gender, age, and open vs. laparoscopic surgery, we cannot exclude the possibility of residual confounding. We were restricted to using the variables available in the registers used. No data were available on BMI, which is a limitation since there is evidence that BMI ≥ 60 is associated with severe psychiatric symptoms and past psychiatric hospitalization [31].

We were only able to identify those with diagnosed psychiatric illness, and we were restricted to the diagnoses made by physicians in hospital inpatient and outpatient care, as diagnoses made by primary care physicians are not included in the National Patient Register. This may have led to an underestimation of the number of patients with depression and SUD, although this should have been negligible given that a filled prescription also was used to identify patients. The cohort was large, but patients with a psychiatric diagnosis have a high rate of refusal for bariatric surgery [32]. Therefore, we were hampered by small sample sizes for some psychiatric diagnoses, such as schizophrenia.

Another limitation is the fact that we did not include laparoscopic sleeve gastrectomy, which is gaining in popularity, nor did we include gastric banding. Neither of these was common in our cohort, and our results are therefore not generalizable to other types of bariatric procedures. Furthermore, there has been a continuous trend towards reduced length of hospital stay for most surgical procedures and an overall reduced rate of complications in recent years. This may affect the generalizability to later cohorts of patients undergoing bariatric surgery.

Delayed discharge and the frequency of reoperation are used as quality metrics of bariatric surgery, and we found a psychiatric diagnosis to be associated with both. To enhance the overall quality of care, further research is needed to better understand the reasons for delayed discharge after GBP surgery in patients with a prior psychiatric diagnosis. It is important to identify strategies and interventions that can reduce the rate of reoperation in the first month after GBP surgery in this patient group. Identification of the patients in need of extra support in coping following surgery is crucial. Devoting extra attention to patient-specific needs and worries that can be addressed to reduce the risk of a prolonged postoperative hospital stay is a key. In this regard, multidisciplinary teams for collaborative patient-centered care [33] may prove to be a fruitful way forward.

## Conclusions

A prior psychiatric diagnosis was found to be associated with delayed discharge following GBP surgery, and a higher likelihood of reoperation in the first 30 days postsurgery, compared to the remaining patients.

## Electronic Supplementary Material

ESM 1 (DOCX 23 kb)

ESM 2 (DOCX 20 kb)
