# Supplementary material for: Risk of Delayed Discharge and Reoperation of Gastric Bypass Patients with Psychiatric Comorbidity—a Nationwide Cohort Study
Source: Obes Surg. 2020 Mar 9;30(7):2511–8. doi: 10.1007/s11695-020-04483-7 (PMC7260256; doi:10.1007/s11695-020-04483-7)
Supplement: Supplementary file 1 — (DOCX 23 kb) [file 11695_2020_4483_MOESM1_ESM.docx]

| Supplemental table 1. Associations between psychiatric diagnoses in the 2 years preceding gastric bypass and delayed discharge after gastric bypass surgery, Odds Ratios (OR) with 95% Confidence Intervals (CI). | | | | | | |
| --- | --- | --- | --- | --- | --- | --- |
|  | Total | Hospital stay  ≥ 90^th^ percentile of number of postoperative days in somatic ward^d^ | | Crude OR conditioned on calendar year  (95% CI) | Model 1^b^  Adjusted OR (95% CI) | Model 2^c^  Adjusted OR (95% CI) |
|  | Number | Number | % |  |  |  |
| All | 22539 | 2215 | 9.8 |  |  |  |
|  |  |  |  |  |  |  |
| None of the below mentioned diagnoses and no antidepressant medication prescribed since 2005 | 15313 | 1341 | 8.8 | REF 1.00 | REF 1.00 | REF 1.00 |
| Diagnosis below or antidepressant medication | 7226 | 874 | 12.1 | 1.39 (1.24-1.56) | 1.53 (1.35-1.73) | 1.48 (1.32-1.67) |
| Diagnosis below | 3200 | 427 | 13.3 | 1.48 (1.32-1.67) | 1.62 (1.43-1.84) | 1.56 (1.39-1.76) |
|  |  |  |  |  |  |  |
| Diagnoses 2 years preceding gastric bypass surgery |  |  |  |  |  |  |
| Severe mental illness (Bipolar disorder/ Schizophrenia)^a^ | 270 | 32 | 11.9 | 1.48 (1.01-2.17) | 1.81 (0.99-3.31) | 1.75 (0.99-3.11) |
| Depression^a , e^ | 1117 | 131 | 11.7 | 1.26 (1.04-1.53) | 1.21 (0.89-1.66) | 1.17 (0.87-1.58) |
| Neurotic disorders^f^ | 2035 | 281 | 13.8 | 1.37 (1.20-1.58) | 1.57 (1.31-1.89) | 1.54 (1.30-1.82) |
| Attention deficit hyperactivity disorder | 251 | 30 | 12.0 | 1.50 (1.00-2.23) | 2.25 (1.17-4.32) | 2.15 (1.16-3.99) |
| Substance use disorder or treatment for substance use disorder | 313 | 42 | 13.4 | 1.53 (1.09-2.14) | 2.46 (1.47-4.11) | 2.49 (1.55-4.00) |
| Eating disorder | 112 | 16 | 14.3 | 1.51 (0.88-2.60) | 2.44 (1.00-5.93) | 1.81 (0.76-4.30) |
| Personality disorder | 309 | 45 | 14.6 | 1.69 (1.22-2.36) | 3.39 (1.69-6.79) | 3.23 (1.69-6.17) |
| Self-harm | 448 | 59 | 13.2 | 1.21 (0.92-1.61) | 1.47 (0.98-2.21) | 1.42 (0.97-2.07) |
|  |  |  |  |  |  |  |
| None of the above, but antidepressant medication prescribed in the 2 years preceding gastric bypass surgery^g^ | 4026 | 447 | 11.7 | 1.30 (1.16-1.46) | 1.27 (1.12-1.44) | 1.27 (1.12-1.44) |
| ^a^ In case of diagnoses of both severe mental illness and depression, severe mental illness was chosen.  ^b^ Adjusted for age, sex, conditioned on calendar year.  ^c^ Adjusted for age, sex, type of gastric bypass surgery, the other diagnoses since 1997 (including treatment for SUD, but not antidepressant medication) and conditioned on calendar year.  ^d^ ≥ 90^th^ percentile of number of postoperative days was ≥6 days in 2008-2009, ≥5 days in 2010, and ≥4 days in 2011-2012.  ^e^ Does not include the group prescribed antidepressants with no diagnosis of depression.  ^f^ Agoraphobia, anxiety disorders, obsessive-compulsive disorder, reaction to severe stress including post-traumatic stress syndrome, adjustment disorders, dissociative and conversion disorders, somatoform disorders, other nonpsychotic mental disorders  ^g^ Filled prescription from the pharmacotherapeutic group N06A (according to the Anatomical Therapeutic Chemical classification system, ATC). | | | | | | |
