# Supplementary material for: Risk of Delayed Discharge and Reoperation of Gastric Bypass Patients with Psychiatric Comorbidity—a Nationwide Cohort Study
Source: Obes Surg. 2020 Mar 9;30(7):2511–8. doi: 10.1007/s11695-020-04483-7 (PMC7260256; doi:10.1007/s11695-020-04483-7)
Supplement: Supplementary file 2 — (DOCX 20 kb) [file 11695_2020_4483_MOESM2_ESM.docx]

Supplemental table 2. Associations between psychiatric diagnoses in the 2 years preceding gastric bypass surgery and reoperation within 30 days of gastric bypass surgery, Odds Ratios (OR) with 95% Confidence Intervals (CI).

|  | Total | Reoperation within 30 days | | Crude OR conditioned on calendar year (95% CI) | Model 1^b^  Adjusted OR (95% CI) | Model 2^c^  Adjusted OR (95% CI) |
| --- | --- | --- | --- | --- | --- | --- |
|  | Number | Number | % |  |  |  |
| All | 22539 | 1270 | 5.6 |  |  |  |
|  |  |  |  |  |  |  |
| None of the below mentioned diagnoses and no antidepressant medication prescribed since 2005 | 15313 | 801 | 5.2 | REF 1.00 | REF 1.00 | REF 1.00 |
| Diagnosis below or antidepressant medication | 7226 | 469 | 6.5 | 1.24 (1.10-1.40) | 1.24 (1.10-1.39) | 1.24 (1.10-1.40) |
| Diagnosis below | 3200 | 215 | 6.7 | 1.28 (1.09-1.49) | 1.35 (1.15-1.58) | 1.30 (1.12-1.52) |
|  |  |  |  |  |  |  |
| Diagnoses in the 2 years preceding gastric bypass surgery |  |  |  |  |  |  |
| Severe mental illness (Bipolar disorder/ Schizophrenia)^a^ | 270 | 17 | 6.3 | 1.20 (0.73-1.98) | 1.12 (0.54-2.36) | 1.14 (0.54-2.39) |
| Depression^a, d^ | 1117 | 60 | 5.4 | 1.02 (0.78-1.34) | 1.04 (0.71-1.53) | 1.05 (0.71-1.55) |
| Neurotic disorders ^e^ | 2035 | 134 | 6.6 | 1.24 (1.03-1.50) | 1.49 (1.19-1.87) | 1.48 (1.18-1.87) |
| Attention deficit hyperactivity disorder | 251 | 10 | 4.0 | 0.77 (0.41-1.46) | 0.46 (0.14-1.48) | 0.46 (0.14-1.51) |
| Substance use disorder or treatment for substance use disorder | 313 | 28 | 8.9 | 1.77 (1.19-2.62) | 2.31 (1.33-4.01) | 2.22 (1.27-3.87) |
| Eating disorder | 112 | 9 | 8.0 | 1.52 (0.76-3.01) | 2.80 (1.07-7.31) | 3.17 (1.21-8.29) |
| Personality disorder | 309 | 23 | 7.4 | 1.45 (0.94-2.22) | 2.29 (1.04-5.01) | 2.28 (1.03-5.05) |
| Self-harm | 448 | 26 | 5.8 | 1.07 (0.71-1.60) | 1.24 (0.74-2.09) | 1.27 (0.75-2.13) |
|  |  |  |  |  |  |  |
| None of the above, but antidepressant medication prescribed in the 2 years preceding gastric bypass surgery ^f^ | 4026 | 254 | 6.3 | 1.18 (1.02-1.36) | 0.92 (0.77-1.11) | 0.92 (0.77-1.11) |
| ^a^ In case of diagnoses of both severe mental illness and depression, severe mental illness was chosen.  ^b^ Adjusted for age, sex, conditioned on calendar year.  ^c^ Adjusted for age, sex, type of gastric bypass surgery, the other diagnoses since 1997 (including treatment for SUD, but not antidepressant medication) and conditioned on calendar year.  ^d^ Does not include the group prescribed antidepressants with no diagnosis of depression.  ^e^ Agoraphobia, anxiety disorders, obsessive-compulsive disorder, reaction to severe stress including post-traumatic stress syndrome, adjustment disorders, dissociative and conversion disorders, somatoform disorders, other nonpsychotic mental disorders  ^f^ Filled prescription from the pharmacotherapeutic group N06A (according to the Anatomical Therapeutic Chemical classification system, ATC). | | | | | | |
